# The cytokine and chemokine expression profile of nucleus pulposus cells: implications for degeneration and regeneration of the intervertebral disc

**DOI:** 10.1186/ar4408

**Published:** 2013-12-11

**Authors:** Kate L E Phillips, Neil Chiverton, Anthony LR Michael, Ashley A Cole, Lee M Breakwell, Gail Haddock, Rowena AD Bunning, Alison K Cross, Christine L Le Maitre

**Affiliations:** 1 Biomedical Research Centre, Sheffield Hallam University, Howard Street, Sheffield, South Yorkshire S1 1WB, UK; 2 Sheffield Teaching Hospitals NHS Foundation Trust, Sheffield, UK

## Abstract

**Introduction:**

The aims of these studies were to identify the cytokine and chemokine expression profile of nucleus pulposus (NP) cells and to determine the relationships between NP cell cytokine and chemokine production and the characteristic tissue changes seen during intervertebral disc (IVD) degeneration.

**Methods:**

Real-time q-PCR cDNA Low Density Array (LDA) was used to investigate the expression of 91 cytokine and chemokine associated genes in NP cells from degenerate human IVDs. Further real-time q-PCR was used to investigate 30 selected cytokine and chemokine associated genes in NP cells from non-degenerate and degenerate IVDs and those from IVDs with immune cell infiltrates (‘infiltrated’). Immunohistochemistry (IHC) was performed for four selected cytokines and chemokines to confirm and localize protein expression in human NP tissue samples.

**Results:**

LDA identified the expression of numerous cytokine and chemokine associated genes including 15 novel cytokines and chemokines. Further q-PCR gene expression studies identified differential expression patterns in NP cells derived from non-degenerate, degenerate and infiltrated IVDs. IHC confirmed NP cells as a source of IL-16, CCL2, CCL7 and CXCL8 and that protein expression of CCL2, CCL7 and CXCL8 increases concordant with histological degenerative tissue changes.

**Conclusions:**

Our data indicates that NP cells are a source of cytokines and chemokines within the IVD and that these expression patterns are altered in IVD pathology. These findings may be important for the correct assessment of the ‘degenerate niche’ prior to autologous or allogeneic cell transplantation for biological therapy of the degenerate IVD.

## Introduction

Intervertebral discs (IVDs) are amphiarthroses that function to permit flexion, extension and lateral bending of the spine. IVDs comprise three distinct tissue regions; the endplates, the annulus fibrosus (AF) and the nucleus pulposus (NP). The NP is the central gelatinous region and consists of a sparse cell population [1] within a complex hydrated extracellular matrix (ECM) of collagen fibres and hydrophilic proteoglycans [2]. The NP is constrained around the periphery by the fibrous concentric lamella of the AF, and above and below by the endplates and the vertebral bodies that each IVD separates.

IVD degeneration is a non-inflammatory arthropathy; as such, the detrimental tissue remodelling events that occur result from alterations in the behaviour of the native cell population. During degeneration, the NP is the site of specific characteristic tissue changes whereby the hydrated gelatinous ECM is condensed and replaced by a more fibrous structure [3]. These structural changes compromise the biomechanics of the IVD, and can lead to prolapse [4].

In degeneration, alterations in native NP cell behaviour occur in respect of proliferation [5], differentiation [6-8], dysregulated metabolism [3,9] and cell death [10,11]. Cytokines are implicated as stimulus for the characteristic alterations in cell behaviour. IL-1 and TNF-α are produced within the IVD, and expression levels are increased in degeneration [12-16]. Further, it is reported that *in vitro* IL-1 or TNF-α stimulation disrupts cellular metabolism in a similar manner to that seen in IVD degeneration [15,17-19]. However, in IVD prolapse, which can occur independently (as a result of exceeding the physiological limit of flexion or compressive force through a motion segment [20,21], or as a secondary complication of IVD degeneration [22], locally produced cytokines may form part of the mechanism of spontaneous resorption. The spontaneous resorption of prolapsed lumbar IVD tissue has frequently been observed [23-25] although the exact mechanisms by which this occur are not yet fully understood. Inflammatory and autoimmune responses provoked in the innate and adaptive immune systems on detection of displaced tissue likely play a role, with infiltrating monocytes [26], macrophages [26-29], T and B lymphocytes [27,28] and fibroblasts [29] reported in prolapsed NP tissue.

To understand the pathogenesis of degeneration, it is important to determine the factors present within the IVD that may elicit behavioural effects on the cells present. Currently, several research initiatives are investigating the regenerative potential of autologous or allogeneic cell transplantation, and mesenchymal or adipose-derived stem-cell transfer to enhance the degenerate native cell population [30-35]. To this end, an understanding of the micro-environmental conditions of the degenerate niche would provide indication as to the factors that may act upon cells, native or introduced, to such an environment. Expression of several cytokines and chemokines (chemo-attractant cytokines) have been identified within the IVD [14,26,36-38], although, with the exception of IL-1 and TNF-α, the cellular source and biological activities of these factors has not been well studied [12,15]. In these investigations, we address the hypothesis that cytokines and chemokines are integral to the pathogenesis of IVD degeneration and prolapse. Specifically, we aimed to identify the cytokine and chemokine expression profile of NP cells and the relationships between cytokine and chemokine production, and the characteristic tissue changes seen during IVD degeneration.

## Methods

### Human IVD tissue samples

Fifty human lumbar IVD tissue samples were obtained for use in this study, either at surgery or post-mortem examination (PM) with informed consent of the patient or relatives (Table 1). Local ethics approval was given for this work by Sheffield Research Ethics Committee (09/H1308/70).

**Table 1:** Tissue donor and sample classification details

**Donor details**					**Sample classification**				
**Ref.**	**Source**	**Age (y)**	**Level**	**Intact**	**cDNA**	**IL-16 IHC**	**CCL2 IHC**	**CCL7 IHC**	**CXCL8 IHC**
1	S	42	L4/L5	No	I	4.0*	4.0*	4.2*	4.0*
2	S	40	L5/S1	Yes	I	7.3*	7.3*	5.7*	7.3*
3	S	25	L4/L5	Yes	D	4	4	4.7	4
4	S	48	L4/L5	Yes	NA	4.0*	4.0*	5.7*	4.0*
5	S	33	L5/S1	Yes	D	NA	NA	NA	NA
6	S	NA	NA	NA	NA	6.0*	6.0*	7.0*	6.0*
7	S	62	L4/L5	Yes	NA	7.5	7.5	7.3	7.5
8	S	32	L5/S1	Yes	N	2.3	2.3	3	2.3
9	S	26	L5/S1	Yes	NA	NA	3.3	NA	3.3
10	S	53	L4/L5	No	NA	7.5*	7.5*	7.5*	7.5*
11	S	40	L5/S1	Yes	NA	7.4	7.4	5.5	7.4
12	S	66	L5/S1	NA	NA	5.7*	5.7*	5.5*	5.7*
13	S	34	L4/L5	Yes	NA	6.2	6.2	7.5	6.2
14	S	NA	NA	Yes	D	NA	NA	NA	NA
15	S	45	L5/S1	Yes	D	NA	NA	NA	NA
16	S	26	L4/L5	Yes	I	NA	NA	NA	NA
17	S	23	L4/L5	Yes	N	NA	NA	NA	NA
18	S	29	L4/L5	Yes	I	NA	NA	NA	NA
19	S	35	L5/S1	No	N	NA	NA	NA	NA
20	S	20	L4/L5	No	I	NA	NA	NA	NA
21	S	39	L5/S1	No	I	NA	NA	NA	NA
22	PM	45	L4/L5	Yes	N	1.8	1.8	2	1.8
23	PM	45	L3/L4	Yes	N	3	3	2.5	3
24	PM	45	L5/S1	Yes	NA	3.5	3.5	3.5	3.5
25	S	48	L4/L5	No	D	9.5	9.5	6	9.5
26	S	26	L5/S1	No	D	11	11	3.5	11
27	S	33	L5/S1	Yes	I	8.5*	8.5*	6.5*	8.5*
28	PM	NA	L1/L2	Yes	NA	10	10	6	10
29	PM	NA	L4/L5	Yes	NA	8	8	6	8
30	PM	NA	L5/S1	Yes	NA	7	7	9	7
31	PM	NA	L3/L4	Yes	NA	8	8	7.3	8
32	PM	NA	L2/L3	Yes	NA	11	11	10.3	11
33	S	42	L5/S1	Yes	N	NA	NA	NA	NA
34	S	36	L5/S1	Yes	D	8	8	4	8
35	S	41	L5/S1	Yes	D	7.5	NA	7.5	NA
36	S	NA	NA	NA	I	6.0*	6.0*	4.7*	6.0*
37	S	44	L5/S1	Yes	I	3.0*	3.0*	2.3*	3.0*
38	S	52	L4/L5	Yes	D	10.5	10.5	7	10.5
39	S	NA	L4/L5	No	I	9.0*	9.0*	6.2*	9.0*
40	S	38	L5/S1	No	I	5.8*	5.8*	5.2*	5.8*
41	S	28	L4/L5	Yes	D	7.7	7.7	3.5	7.7
42	S	43	L5/S1	No	D	NA	NA	NA	NA
43	S	44	L5/S1	Yes	D	NA	NA	NA	NA
44	S	28	L5/S1	No	D	NA	NA	NA	NA
45	S	35	L5/S1	Yes	D	NA	NA	NA	NA
46	S	43	L5/S1	No	D	NA	NA	NA	NA
47	S	42	L5/S1	No	D	NA	NA	NA	NA
48	S	62	L3/L4	Yes	I	NA	NA	NA	NA
49	S	40	L3/L4	Yes	D	NA	NA	NA	NA
50	S	45	L4/L5	No	I	NA	NA	NA	NA

S, surgical; PM, post-mortem; I, infiltrated; D, degenerate; N, non-degenerate, IHC, immunohistochemistry; CCL, C-X-C chemokine. Surgically obtained prolapsed tissue samples were considered to be from intact IVDs when no signs of annulus fibrosus or cartilaginous endplate rupture were evident (protrusion-type prolapse). Values given for IHC sample classification are average histologically determined grades of degeneration; ^*^infiltrating immune cells were observed; NA, not applicable.

#### PM tissue

Eight IVDs were recovered from two donors with no recorded history of IVD disease or low back pain. They consisted of whole IVDs from which full-thickness wedges of 120° of arc were removed anteriorly for processing to paraffin wax. Remaining NP tissue was washed in sterile phosphate-buffered saline and placed for NP cell isolation.

#### Surgical tissue

Forty-two NP samples were recovered from patients undergoing microdiscectomy surgery for alleviation of root pain related to IVD prolapse at Northern General Hospital, Sheffield, UK. Surgical samples consisted of multiple fragments of NP tissue with some samples containing fragments of NP with AF or cartilaginous endplate (CEP) attached. All surgical samples were assessed macroscopically upon receipt at the processing laboratory; where possible, one large fragment of NP only was divided into two, with one half being processed to paraffin wax and the other half placed for cell isolation; any remaining smaller fragments were also processed to paraffin wax. Samples containing multiple small NP fragments or a mixture of small and large NP fragments where the large fragments had AF or CEP attached were processed in their entirety to paraffin wax.

### Processing to paraffin wax, routine histology and immunohistochemistry for sample grading

Tissue samples were fixed in 10% neutral buffered formalin (Leica, Milton Keynes, UK) and processed to paraffin wax on a Shandon Elliott Duplex Processor. Haematoxylin and eosin stained sections were evaluated independently by two researchers (KLEP and CLLM) to determine the extent of degenerative tissue changes. Sections were scored numerically between 0 and 12 based on the presence of cell clusters, fissures, loss of demarcation and haematoxophilia (indicating reduced proteoglycan content); a score of 0 to 3 indicates a histologically normal (non-degenerate) IVD and a grade ≥4 indicates evidence of degeneration, as described previously [15]. Inter-observer scores were averaged and assigned to each tissue sample. Sections were also taken for routine immunohistochemical (IHC) analysis of CD11b expression. CD11b is a leukocyte adhesion molecule and was used to identify immune cell infiltrates in NP tissue samples from prolapsed IVDs. CD11b IHC analysis was performed as described in the IHC method below, using a rabbit polyclonal primary antibody (1:50). Samples were classified as infiltrated on the basis of CD11b immunopositivity.

#### Grade assignment for cDNA samples

On account of the heterogeneity often observed in prolapsed IVD tissues [39], serial sections were made from paraffin-embedded tissues that had matched cDNA samples derived from surgically obtained NP. Routine histological and IHC examinations were made on multiple sections, at different levels throughout the paraffin-embedded tissue, to ensure assessment of any heterogeneity. cDNA samples were only considered to be from non-degenerate tissue if all haematoxylin and eosin stained sections were assigned a histological grade of degeneration <4. cDNA samples were considered to be from an infiltrated IVD if CD11b immunopositivity (indicating leukocyte presence) was observed in any tissue section evaluated.

#### Grade assignment for immunohistochemistry samples

For each tissue section used in IHC evaluation, routine histology was performed on an adjacent serial section. By this method, the grade of degeneration assigned is relevant to the tissue assessed by IHC even in surgically obtained tissue samples where heterogeneity is sometimes observed.

### Cell isolation and generation of cDNA samples

NP tissue samples were finely minced and digested with 2 U/ml protease (Sigma, Poole, UK) in DMEM (Gibco, Paisley, UK) for 30 minutes at 37°C, followed by 2 mg/mL collagenase type I (Sigma) in DMEM for 4 hours at 37°C. Cells were recovered by centrifugation and RNA extracted immediately from 1 × 10^5^ cells using tri-reagent (Invitrogen, Paisley, UK). Genomic DNA contamination was eliminated by DNase treatment (Qiagen, Crawley, UK) prior to RNA purification by MinElute Cleanup kit (Qiagen) as per manufacturer's protocol. cDNA was reverse transcribed using Moloney Murine Leukaemia Virus reverse transcriptase (Bioline, London, UK) and random hexamers (Applied Biosystems, Warrington, UK).

### CDNA low density array

**Figure 1:**
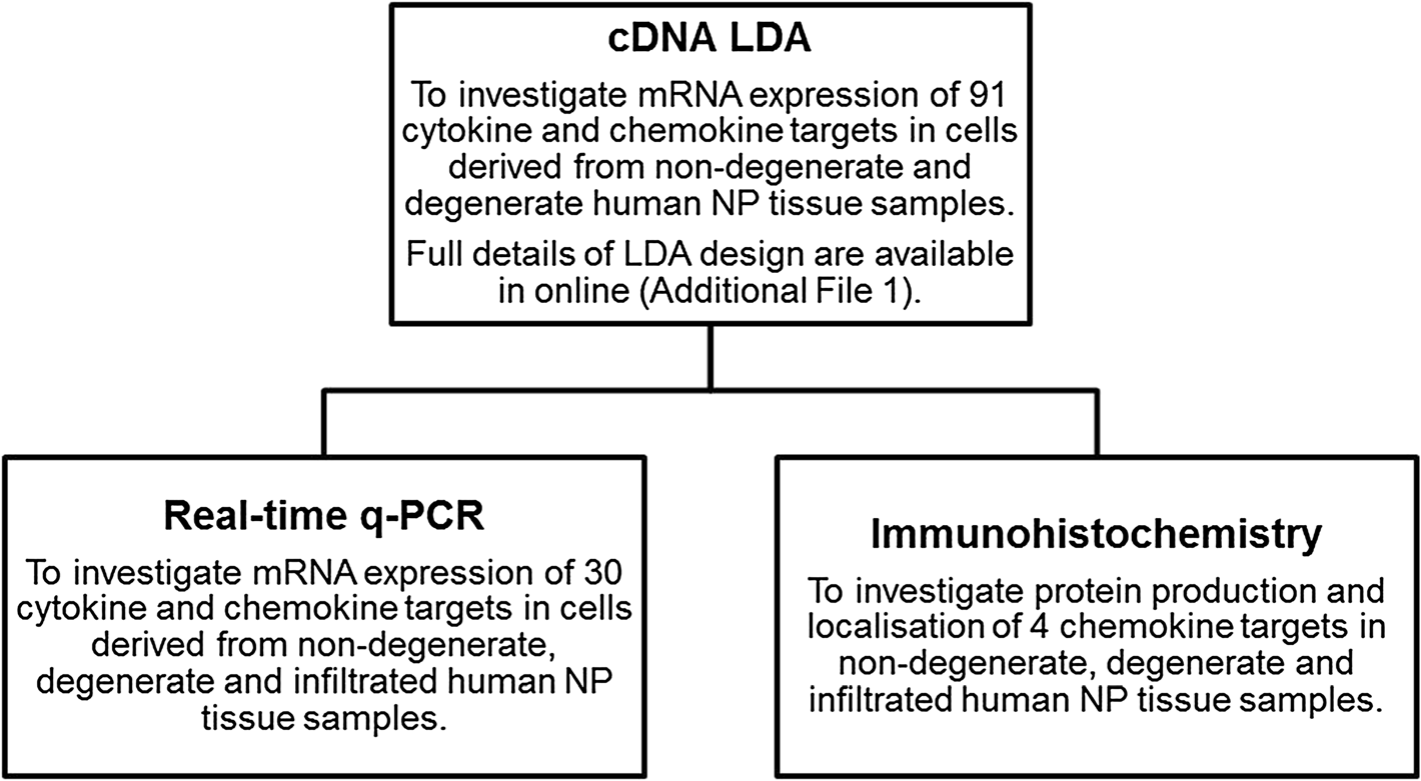
**Study outline and experimental approaches.** Initial low density array (LDA) analysis of cells isolated from human nucleus pulposus (NP) tissue samples identified a diverse cytokine and chemokine expression profile. From these data, 30 targets were selected for further gene expression analysis by real-time qPCR and 4 targets were selected for protein production and localisation analysis by immunohistochemistry. Consideration was given to the frequency at which targets were detected and any differential expression profile observed between non-degenerate and degenerate study groups to select targets for further investigation. Consideration was also given to the detection of receptor mRNA expression (indicating potential for autocrine or paracrine signalling involvement), whether the roles of identified targets had previously been well studied, the identification of novel targets within the intervertebral disc, and links to cytokine and chemokine activity in other arthropathies.

In total, six cDNA samples were interrogated by real-time PCR low density array (LDA; Applied Biosystems) for the expression of 91 cytokine- and chemokine-associated genes. Three cDNA samples were selected from each of two study groups; non-degenerate (mean age 34 years, range 25 to 45) and degenerate (mean age 32 years, range 29 to 33), according to routine histological examination of matched paraffin-embedded tissues. Briefly, a mastermix was prepared for each sample and 20 μL per well loaded onto 96-well FAST® LDA plates - equivalent to 2 μL cDNA, 10 μL Taqman® FAST® universal mastermix (Applied Biosystems) and 8 μL sterile deionised water per well. Plates were run on a StepOnePlus real-time PCR machine (Applied Biosystems) on a FAST® programme incorporating 50 cycles of denaturation at 95°C for 1 second followed by annealing and extension at 60°C for 20 seconds. Measurements were performed in duplicate, averaged and normalised to expression of five internal reference genes. Full details of LDA plate design are available online (see Additional file 1). Following analysis of cDNA, LDA data-targets were selected for further investigation by real-time PCR and IHC (Figure 1).

### Real-time PCR

Following LDA analysis 30 cytokine- and chemokine-associated genes were selected for further investigation by real-time q-PCR. In total, 35 cDNA samples (Table 1) were divided into three study groups; non-degenerate, degenerate and infiltrated according to routine histological and IHC examination of matched paraffin-embedded tissues, and subjected to gene expression analysis. Briefly, a mastermix was prepared for each target gene investigated and 8 μL per well loaded onto 96-well FAST® PCR plates - equivalent to 5 μL Taqman® FAST® universal mastermix (Applied Biosystems), 2.5 μL sterile deionised water and 0.5 μL Taqman® gene expression assay (Applied Biosystems) per well, alongside 2 μL sample cDNA. Plates were run on a StepOnePlus real-time PCR machine as described above. Measurements were performed in duplicate, averaged and normalised to expression of two internal reference genes (*GAPDH* and *18S*). Full details of Taqman® gene expression assays used are available online (see Additional file 1).

### Immunohistochemistry

Four chemokines were selected for IHC investigation of protein production and localisation following LDA analysis of gene expression. Expression of each target chemokine was investigated in 30 IVD tissue samples (Table 1), divided between three study groups; non-degenerate, degenerate and infiltrated, according to routine histological and IHC examination. Briefly, 4-μm paraffin sections were de-waxed, rehydrated and endogenous peroxidase-blocked using hydrogen peroxide. After washing in tris-buffered saline (TBS; 20 mM tris, 150 mM sodium chloride, pH 7.5), sections were subjected to heat-induced antigen retrieval (10-minute microwave irradiation in 0.05 M tris buffer, pH 9.5). Following TBS washing, non-specific binding sites were blocked at room temperature for 90 minutes with 25% w/v goat serum (Abcam, Cambridge, UK) in 1% w/v bovine serum albumin in TBS. Sections were incubated overnight at 4°C with rabbit polyclonal primary antibodies against human IL-16 (1:750), CCL2 (1:500), CCL7 (1:10,000) and CXCL8 (1:100). Negative controls in which rabbit IgGs (Abcam) replaced the primary antibody at an equal protein concentration were used. After washing in TBS, sections were incubated in biotinylated goat anti-rabbit antiserum (1:500; Abcam) for 30 minutes at room temperature. Disclosure of secondary antibody binding was by the streptavidin-biotin complex (Vector Laboratories, Peterborough, UK) technique with 0.08% v/v hydrogen peroxide in 0.65 mg/mL 3,3'-diaminobenzidine tetrahydrochloride (Sigma) in TBS. Sections were counterstained with Mayer’s Haematoxylin (Leica), dehydrated, cleared and mounted in Pertex (Leica).

All slides were visualised using an Olympus BX60 microscope and images captured using a digital camera and software program QCapture Pro v8.0 (MediaCybernetics, Marlow, UK). Evaluation of IHC staining was performed by counting 200 NP cells, with immunopositive cells expressed as a percentage of total count. A polarizing filter was used during slide evaluation to ensure cells counted were within NP tissue only, as any AF fragments can be identified by differential collagen-fibre arrangement visible under polarized light. Since the majority of samples collected for use in this study consisted of NP tissue only, no attempt was made to quantify immunopositivity in AF or CEP IVD regions.

### Statistical analysis

Statistical analysis of LDA and q-PCR data was performed against two parameters; where mRNA expression was detected, the Mann-Whitney test (for LDA) or Kruskal-Wallis combined with the Conover-Inman post hoc test (for q-PCR) was used to investigate significant differences in expression level between study groups; when the frequency at which expression was detected was not equivalent between study groups, the two-sample test of proportionality was used to investigate significant differences in detection frequency. Statistical analysis of IHC data was performed against two parameters; the Kruskal-Wallis test was used to investigate significant differences in expression level between study groups, and linear regression analysis was used to investigate correlations between measured protein expression and the extent of degenerative tissue changes.

## Results

### A diverse cytokine and chemokine mRNA expression profile was identified in total cells isolated from human NP tissue specimens

**Figure 2:**
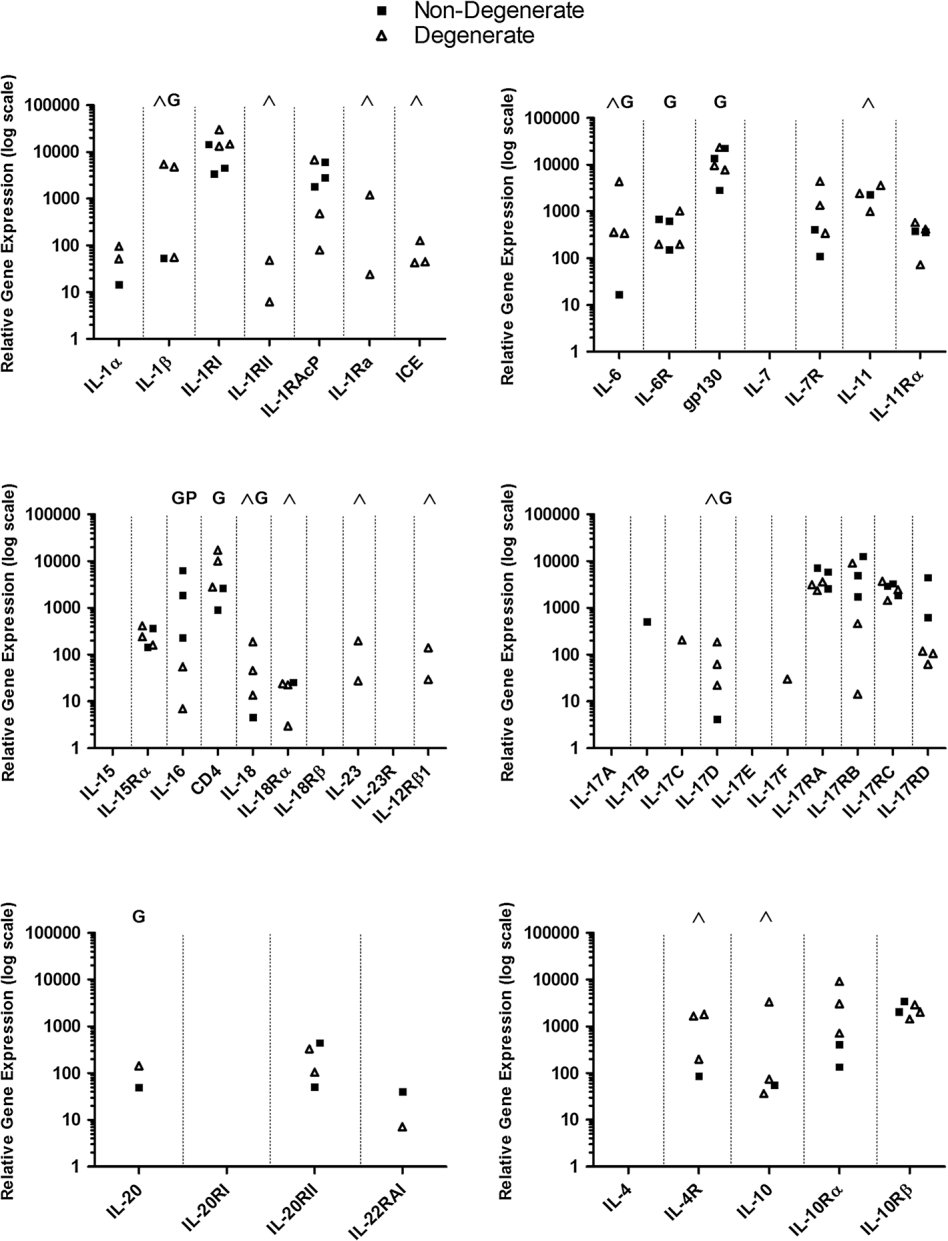
**cDNA low density array cytokine and chemokine expression profile of NP cells.** Gene expression of 36 cytokines and chemokines was detected in nucleus pulposus cells; 15 of the identified cytokines and chemokines have not been identified previously within the intervertebral disc (IVD). Comparative analysis indicates expression of 16 targets is increased in NP cells derived from degenerate IVDs. ^Increased frequency of mRNA detection in NP cells derived from non-degenerate IVDs compared to degenerate (*P* <0.05); G, further gene expression analysis performed on this target; P, further protein expression analysis performed on this target.

**Figure 3:**
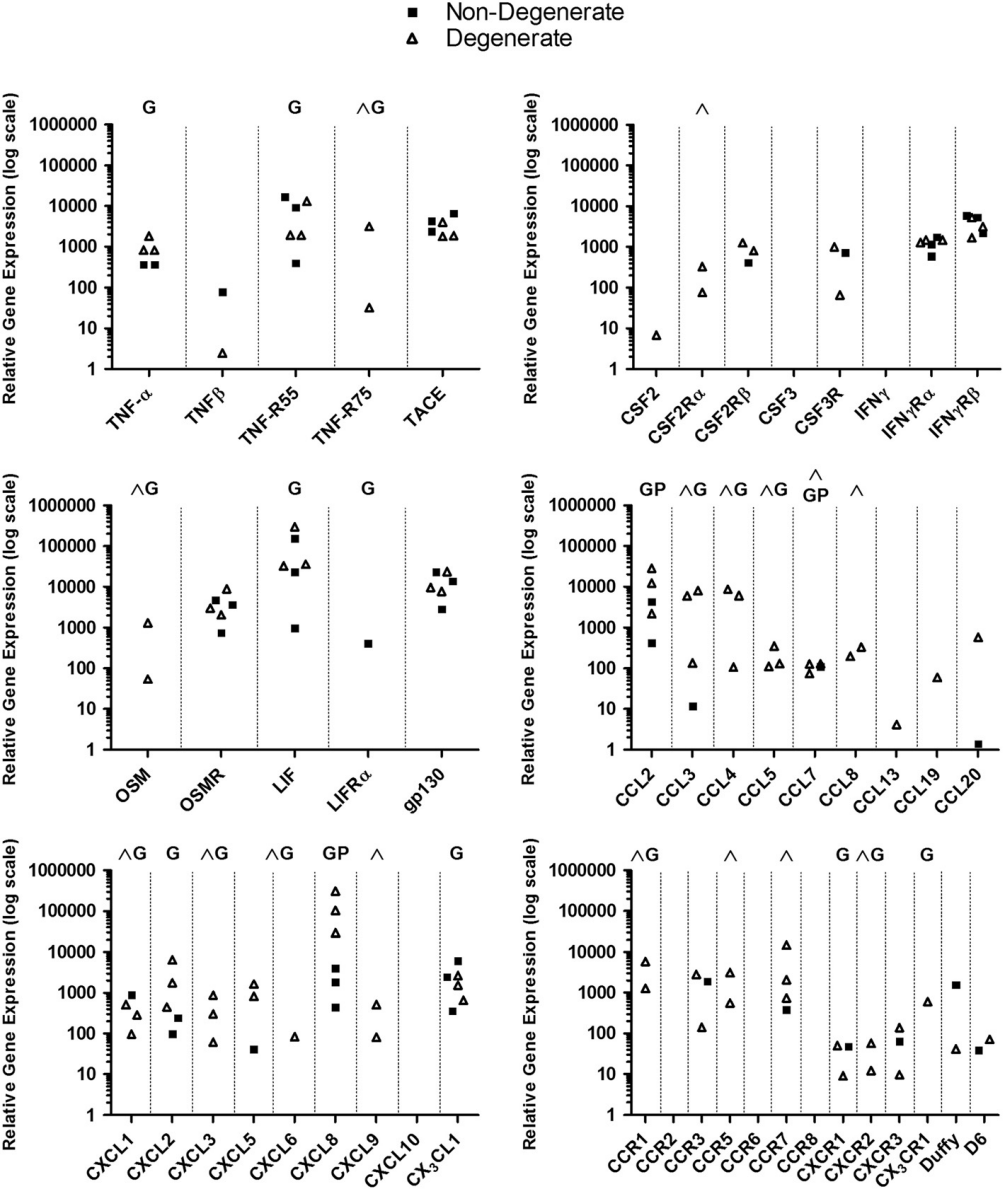
**cDNA low density array LDA cytokine and chemokine expression profile of nucleus pulposus (NP) cells.** Gene expression of 36 cytokines and chemokines was detected in NP cells; 15 of the identified cytokines and chemokines have not been identified previously within the intervertebral disc (IVD). Comparative analysis indicates expression of 16 targets is increased in NP cells derived from degenerate IVDs. ^Increased frequency of mRNA detection in NP cells derived from non-degenerate IVDs compared to degenerate (*P* <0.05); G, further gene expression analysis performed on this target; P, further protein expression analysis performed on this target.

cDNA LDA was performed to assess the diversity and extent of cytokine- and chemokin-associated gene expression by cells isolated from NP tissue. An extensive gene expression profile was detected, including 15 cytokines and chemokines not previously identified within the *IVD; IL-11, IL-16, IL-18, IL-23, TNF-β, OSM, LIF, CCL8, CCL19, CCL20, CXCL2, CXCL3, CXCL5, CXCL6* and *CX*_*3*_*CL1* (Figures 2 and 3). Expression of numerous receptor and signalling accessory molecules was also identified. Comparative analysis of data generated from non-degenerate and degenerate samples suggests that 16 of the identified cytokines and chemokines: *IL-1β, IL-6, IL-10, IL-11, IL-17D, IL-18, IL-23, OSM, CCL3, CCL4, CCL5, CCL7, CCL8, CXCL1, CXCL3* and *CXCL9*, are up-regulated in degenerate samples (Figures 2 and 3).

**Figure 4:**
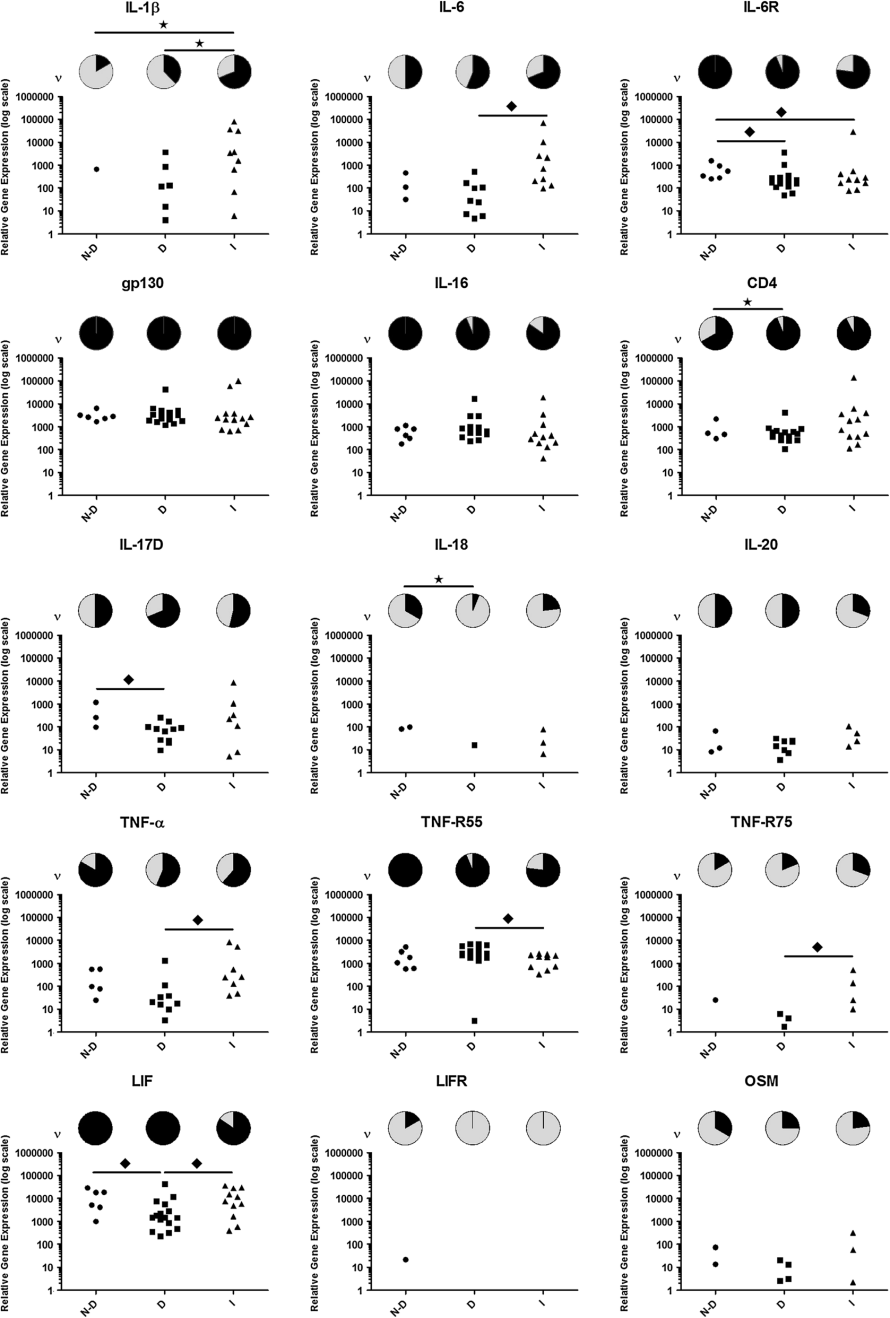
**Cytokine-associated gene expression in nucleus pulposus (NP) cells from non-degenerate, degenerate and infiltrated intervertebral discs (IVDs).** Relative mRNA quantitation of cytokines and receptors was assessed by real-time q-PCR in cDNA samples from non-degenerate, degenerate and infiltrated study groups. Data shown are detection frequency (ν; pie charts) and relative expression per sample investigated (scatter plots). N-D, non-degenerate study group; D, degenerate study group; I, infiltrated study group; black star, significant difference in detection frequency between study groups (*P* <0.05); black diamond, significant difference in relative expression levels between study groups (*P* <0.05). LIFR, leukaemia inhibitory factor receptor.

**Figure 5:**
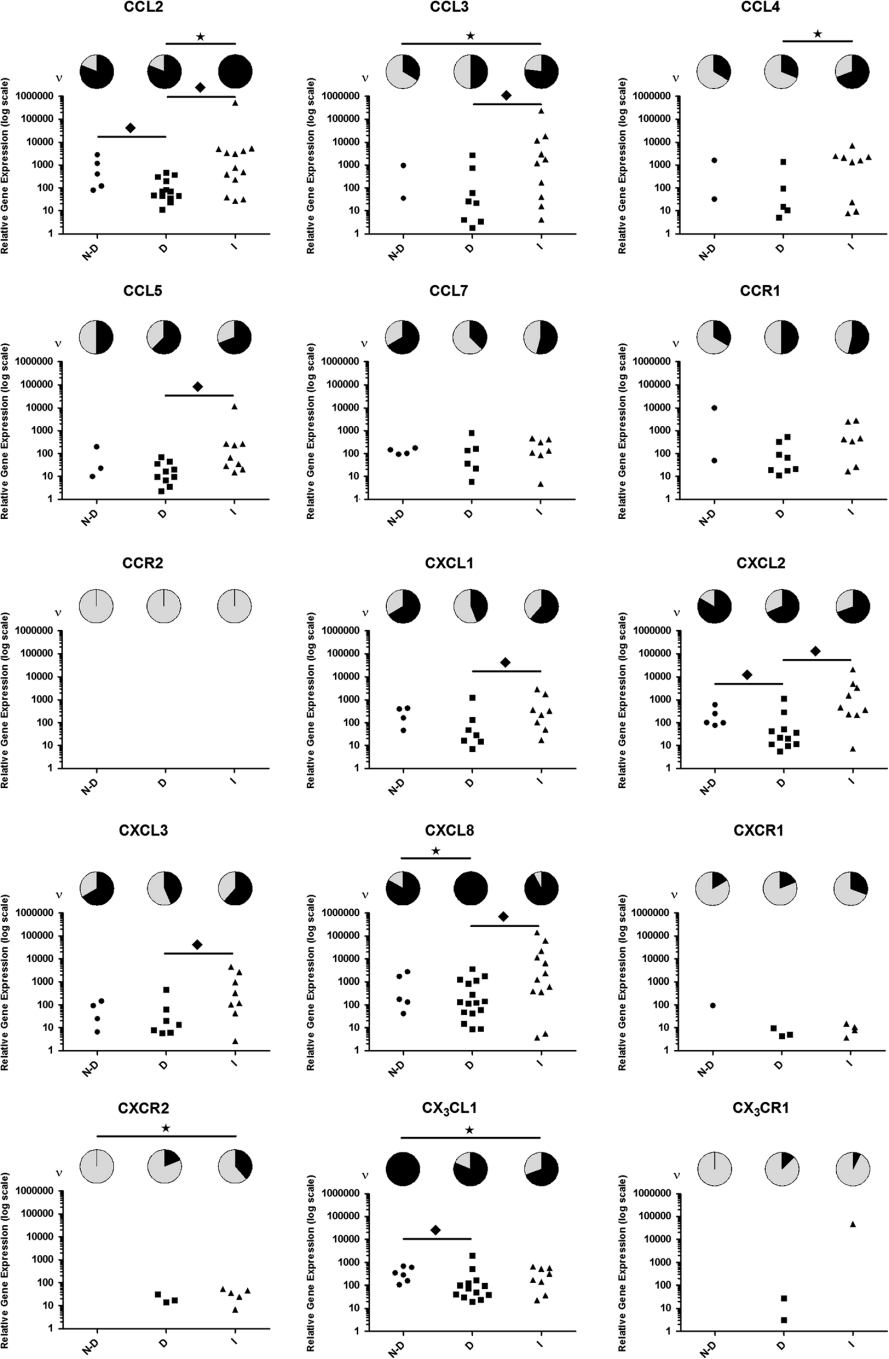
**Chemokine-associated gene expression in nucleus pulposus (NP) cells from non-degenerate, degenerate and infiltrated intervertebral discs (IVDs).** Relative mRNA quantitation of chemokines and receptors was assessed by real-time q-PCR in cDNA samples from non-degenerate, degenerate and infiltrated study groups. Data shown are detection frequency (ν; pie charts) and relative expression per sample investigated (scatter plots). N-D, non-degenerate study group; D, degenerate study group; I, infiltrated study group; black star, significant difference in detection frequency between study groups (*P* <0.05); black diamond, significant difference in relative expression levels between study groups (*P* <0.05).

For thirty cytokine- and chemokine-associated genes identified by LDA, investigations were extended to incorporate 35 cDNA samples across three study groups: non-degenerate, degenerate and infiltrated. Expression of all cytokines and chemokines investigated was detected in all study groups (Figures 4 and 5). In most cases, expression of cytokine and chemokine receptors were detected across study groups, with the exceptions of *LIFR*, which was only detected within non-degenerate samples, *CXCR2* and *CX*_*3*_*CR1*, which were only detected in degenerate and infiltrated samples and *CCR2*, which was not detected in any NP cell sample investigated (Figures 4 and 5).

To ascertain if differential cytokine and chemokine expression profiles are associated with IVD degeneration or are attributable to immune-cell infiltration, comparative analysis was performed across study groups. Differential expression profiles were observed in relation to both the relative level of mRNA expression and frequency at which mRNA expression was detected. Relative expression levels of *IL-17D, LIF* (Figure 4), *CCL2, CXCL2* and *CX*_*3*_*CL1* (Figure 5) were decreased in degenerate samples compared to non-degenerate (*P <*0.05 in all cases) while *CXCL8* expression was detected more frequently in degenerate samples compared to non-degenerate (Figure 5; *P* <0.05). *IL-6* and *IL-16* mRNA expression was equivalent between non-degenerate and degenerate study groups although the relative expression level of IL-6 receptor (*IL-6R*) was decreased in degenerate samples, whereas detection frequency of *CD4* (IL-16 receptor) was increased (Figure 4; *P* <0.05 in both cases).

*IL-1β* (Figure 4) and *CCL3* (Figure 5) expression was detected more frequently in infiltrated samples compared to non-degenerate, whereas *CX*_*3*_*CL1* was detected less frequently in infiltrated samples compared to non-degenerate (*P* <0.05 in all cases). A similar pattern to that seen in degenerate samples was observed in infiltrated samples for *IL-6R* expression (Figure 4), where relative expression level was decreased compared to non-degenerate samples (*P* <0.05). *CXC* chemokine expression was not significantly altered between non-degenerate and infiltrated samples although detection frequency of receptor *CXCR2* was increased in infiltrated samples compared to non-degenerate (Figure 5; *P* <0.05).

Comparative analysis was also performed between the two pathological study groups. Overall, greater cytokine and chemokine expression was detected in infiltrated samples compared to degenerate. *IL-1β* (Figure 4), *CCL2* and *CCL4* (Figure 5) were detected more frequently in infiltrated samples and expression levels of *IL-6, TNF-α, TNF-R75, LIF, CCL2, CCL3, CCL5, CXCL1, CXCL2, CXCL3* and *CXCL8* were greater in infiltrated samples (*P* <0.05 in all cases).

### Quantitation of chemokine production by NP cells in human NP tissue specimens

**Figure 6:**
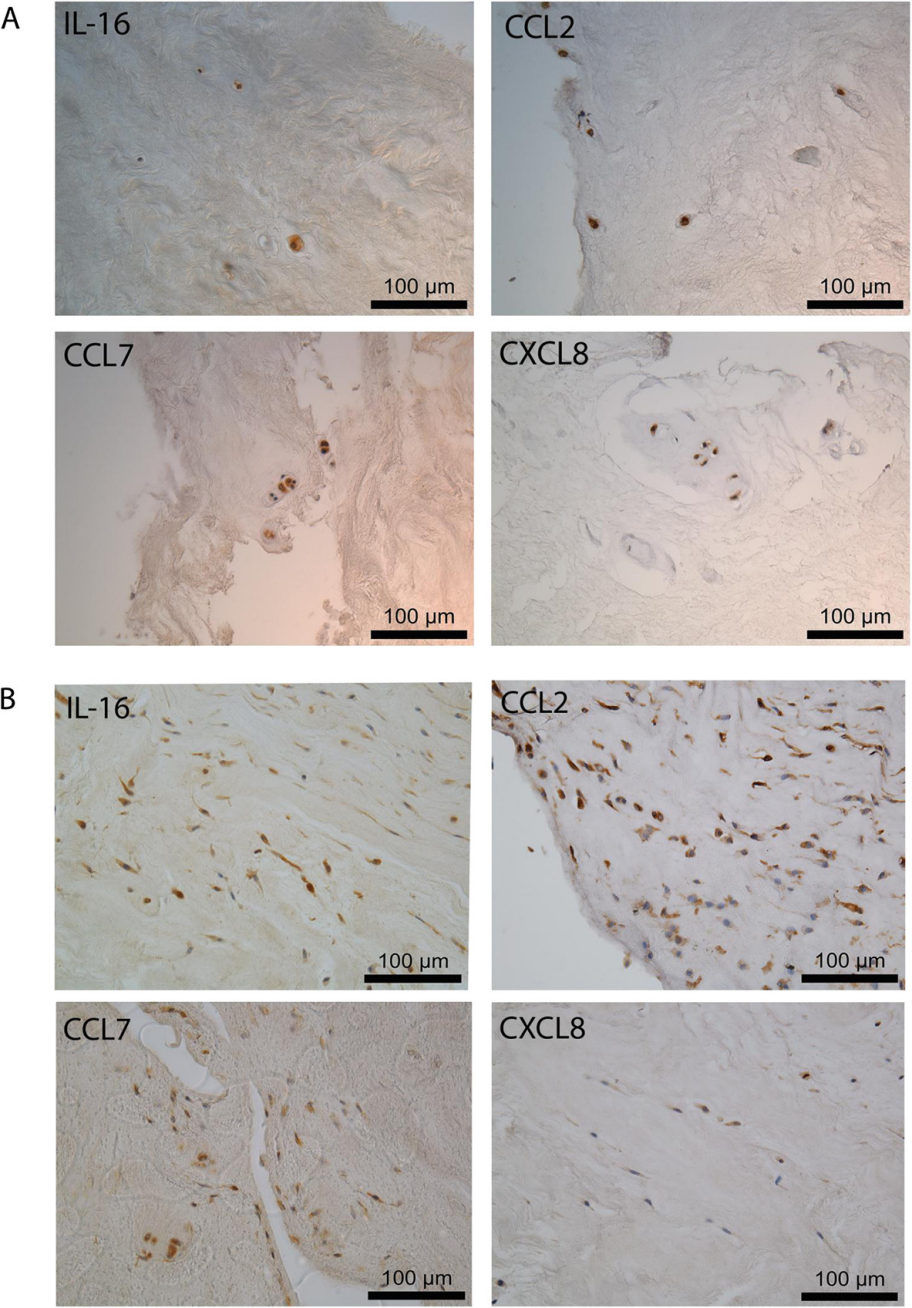
**IL-16, CCL2, CCL7 and CXCL8 expression in nucleus pulposus (NP) tissue from prolapsed intervertebral discs (IVDs).** Representative photomicrographs showing 4-μm paraffin-embedded sections of NP tissue stained for IL-16, CCL2, CCL7 and CXCL8 **(A)**. Abundant IL-16, CCL2, CCL7 and CXCL8 expression was observed in NP cells scattered throughout tissue sections (original magnification ×400, scale bar represents 100 μm). IL-16, CCL2, CCL7 and CXCL8 expression was also observed in immune cell infiltrates **(B)**.

**Figure 7:**
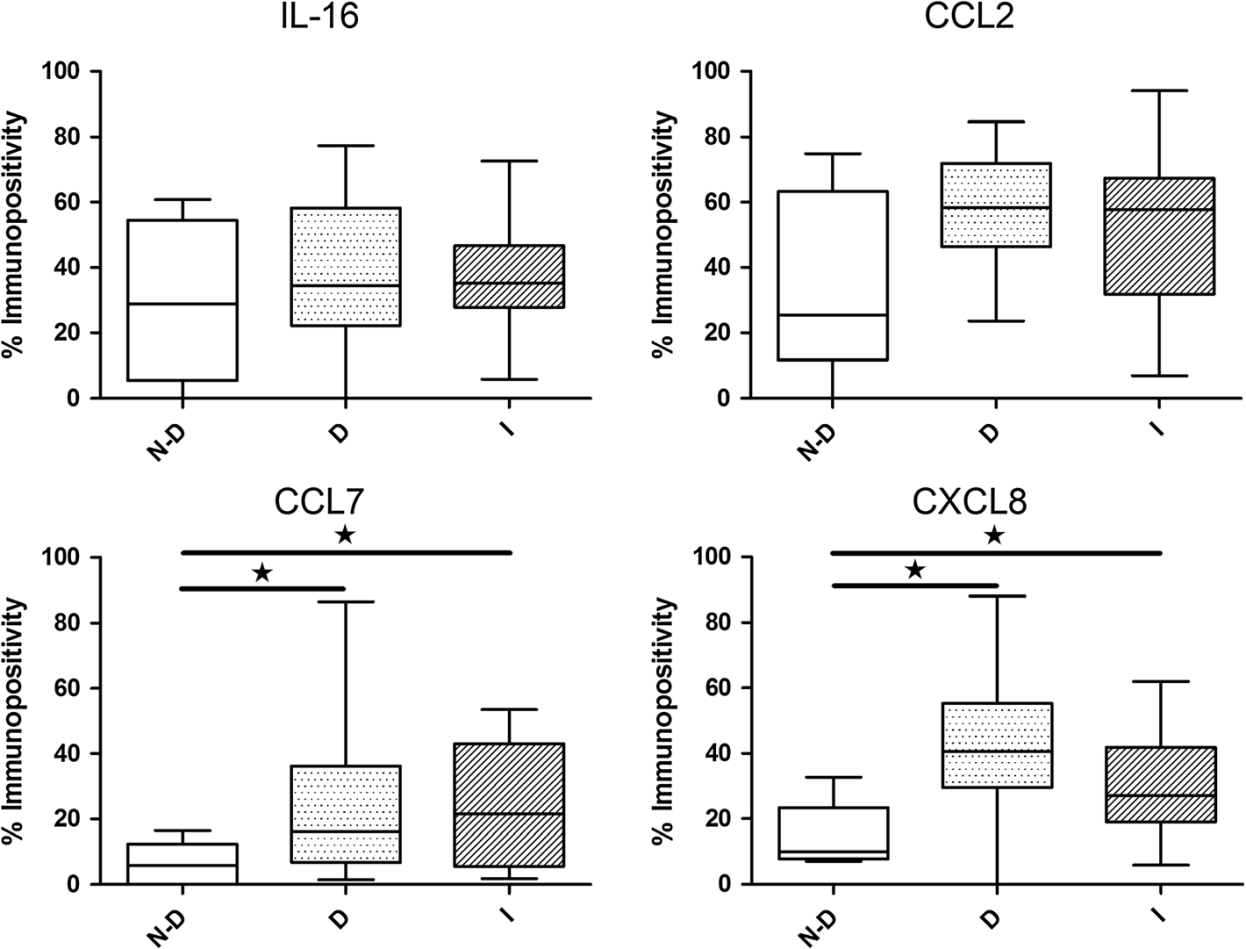
**IL-16, CCL2, CCL7 and CXCL8 expression in non-degenerate, degenerate and infiltrated intervertebral discs (IVDs).** Immunohistochemical quantification of IL-16, CCL2, CCL7 and CXCL8 expression by NP cells in non-degenerate (ND), degenerate (D) and infiltrated (I) IVDs. Data shown are median values for immunopositivity; black star, significant difference in expression between study groups (*P* <0.05). IL-16 expression was equivalent across study groups, CCL2, CCL7 and CXCL8 expression was increased in degenerate samples, CCL7 and CXCL8 expression was also increased in infiltrated samples.

To confirm that gene expression translates to protein production *in vivo*, expression of IL-16, CCL2, CCL7 and CXCL8 in human NP tissue was assessed by IHC. Protein production was confirmed and localised to native NP cells (Figure 6A). Although for all targets investigated, expression was also observed in immune cell infiltrates, where present, in NP tissue specimens, indicating that NP cells are not the only source of cytokines and chemokines within the IVD (Figure 6B). Assessment of percentage cellular immunopositivity localised to NP cells was used to identify differential expression profiles between study groups (Figure 7). Protein expression of CCL7 and CXCL8 localised to NP cells was increased in degenerate samples compared to non-degenerate samples (*P* <0.05 in both cases). Protein expression of CCL2 localised to NP cells was also increased, but not significantly so (*P* = 0.0526), in degenerate samples compared to non-degenerate. Expression of CCL7 and CXCL8 localised to NP cells was increased in infiltrated samples compared to non-degenerate (*P* <0.05 in both cases). Expression of CCL2 localised to NP cells was also increased, but not significantly so (*P* = 0.0870), in infiltrated samples compared to non-degenerate. IL-16 expression was equivalent across all study groups.

**Figure 8:**
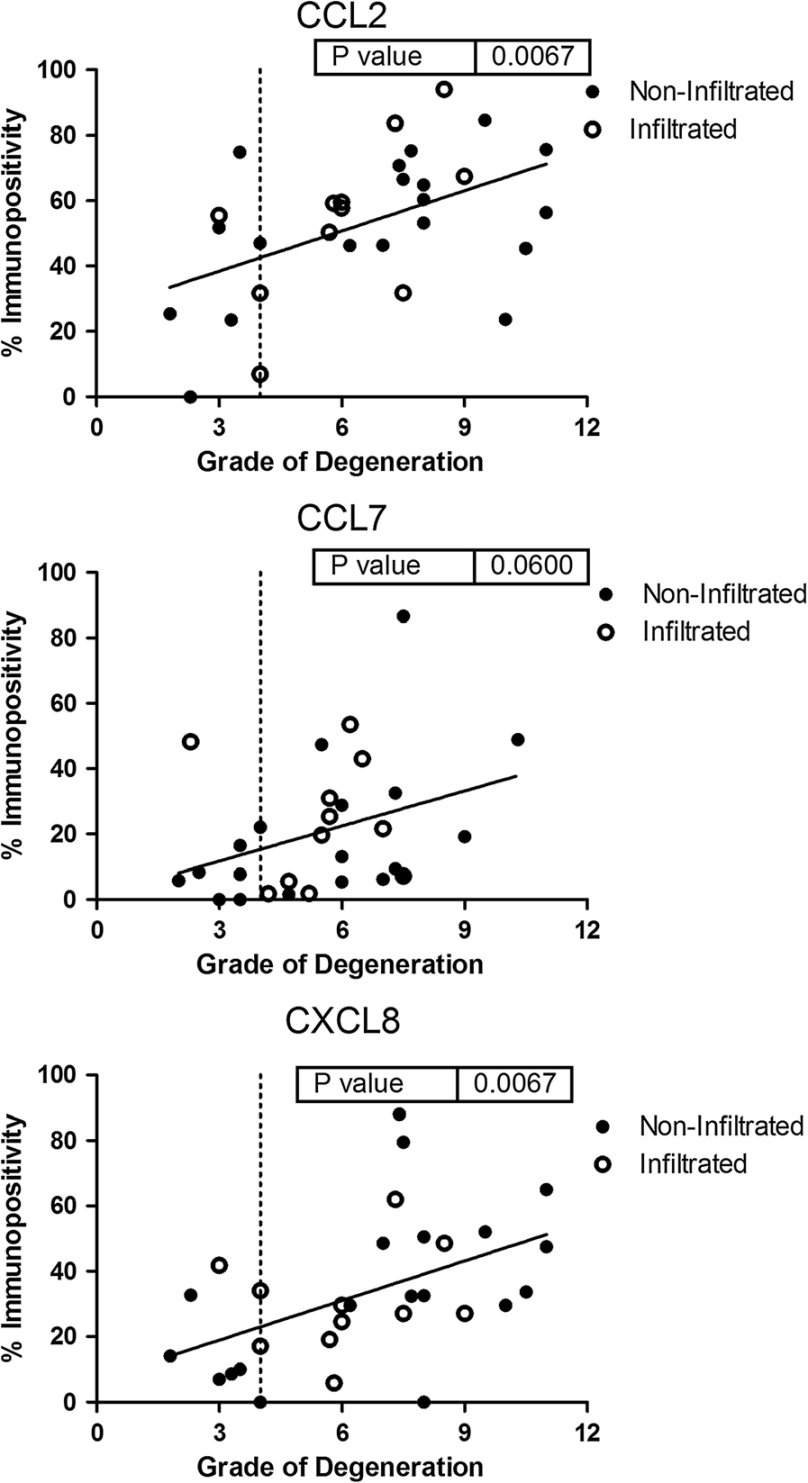
**CCL2, CCL7 and CXCL8 expression correlates with degenerative tissue changes.** Immunohistochemical quantification of CCL2, CCL7 and CXCL8 expression by nucleus pulposus cells. Data shown as are immunopositivity values against histologically determined grade of degeneration. Linear regression analysis was performed to assess correlation between histologically determined grade of degeneration and immunopositivity.

We then examined if degenerative tissue changes correlated with NP cell protein expression of cytokines and chemokines localised to NP cells, as determined by IHC (Figure 8). Protein expression of CCL2 and CXCL8 by NP cells increased concordant to increasing severity of degenerative tissue changes in human NP (*P* = 0.0067 and *P* = 0.0067 respectively). Protein expression of CCL7 also increased concordant to degenerative tissue changes, but not significantly so (*P* = 0.0600).

## Discussion

In the last decade, our knowledge of cytokine effects within the IVD has increased substantially, particularly in respect of IL-1 and TNF-α and the detrimental effects of elevated levels of these cytokines [12,13,15-19,37,40]. On the contrary, the full extent of cytokine and chemokine production by native IVD cells has not been fully explored [14,26,36,38]. To the best of our knowledge, the current study is the first to systematically profile cytokine and chemokine expression in NP tissue.

In cells isolated from degenerate human tissue we observed an extensive cytokine and chemokine expression profile. Cytokine and chemokine ligands were detected in most cases alongside receptors, activating enzymes and signalling accessory proteins, thus there is the potential for these ligands to act as autocrine or paracrine factors on the native NP cell population within the IVD. The effects of IL-1 and TNF-α on NP cell behaviour have been investigated previously, with both factors eliciting effects on cellular metabolism [15,17-19], a parameter that is adversely affected in IVD degeneration [3,9,41]. Notably, it is not only the expression of IL-1 and TNF-α that is increased in the degenerate IVD [13,15]; the cellular response to stimulation is altered [15,18], as is the sensitivity of the cell population, evidenced by altered expression patterns of the receptors [13,15,16]. Further, imbalance between IL-1 and its natural inhibitor, IL-1 receptor antagonist, is reported to induce spontaneous IVD degeneration in mice [40]. Although IL-1 and TNF-α are implicated in the pathogenesis of IVD degeneration, both are expressed at low levels in the normal IVD, indicating a role for these factors under normal physiological conditions [12,13,15,16].

Additional gene expression studies confirmed that the cytokine- and chemokine-associated gene expression profile observed in cells from degenerate IVDs was similar to that from non-degenerate and infiltrated IVDs, reinforcing the potential of these factors to act as intercellular signalling molecules under normal physiological conditions and in the pathologies of IVD degeneration and prolapse. Although expression of the same factors was identified, comparative analysis indicated that differential expression patterns exist in relation to detection frequency and relative expression levels. Within the non-degenerate and degenerate study groups mRNA detection is assumed to be a result of native NP cell expression. Interestingly, at the level of mRNA detection, relative expression of several cytokine and chemokine ligands; *IL-17D, LIF, CCL2, CXCL2* and *CX*_*3*_*CL1* was down-regulated in degeneration. Only *CXCL8* mRNA expression was significantly up-regulated in degenerate samples compared to non-degenerate counterparts. In the infiltrated study group, mRNA expression may result from a combination of NP and infiltrating immune cell expression. Generally, in infiltrated samples cytokine- and chemokine-associated gene expression was increased compared to both non-degenerate and degenerate samples, particularly so for *IL-1β, IL-6, TNF-α, CCL3*[37] and *CXCL8* and this may be due to summated expression by native NP and infiltrating immune cells.

IHC studies confirmed that a source of cytokine and chemokine production in the IVD is the native NP cell population in respect of IL-16, CCL2, CCL7 and CXCL8, and that production is a feature of NP cells in non-degenerate and pathological IVD tissues. This agrees with previous reports of NP cell production of IL-1 [15,16], CCL3 and CCL4 [37]. Comparative analysis of NP cell production in non-degenerate, degenerate and infiltrated IVDs shows that protein expression of CCL2, CCL7 and CXCL8 is increased in degenerate and infiltrated IVDs compared to the non-degenerate counterpart. At the level of NP cell immunopositivity, no significant differences between degenerate and infiltrated study groups were observed, whereas previous gene expression analysis indicated that CCL2 and CXCL8 mRNA expression was greater in infiltrated compared to degenerate samples. This finding would again point towards infiltrating immune cells as a contributing source of CCL2 and CXCL8 expression within the IVD, as gene expression was measured in total cells isolated from tissue specimens, and IHC identified expression of IL-16, CCL2, CCL7 and CXCL8 in immune cell infiltrates, although this was not quantified. Of note, upon histological examination of tissue sections, immune-cell infiltrates were identified in tissue samples from clinically reported intact IVDs (protrusion-type prolapse). As leukocyte infiltration usually occurs following AF or CEP rupture [27-29,42] and exposure of NP tissue to the external environment of the IVD [28,43-47], a possible explanation for this finding is that NP tissue displaced down a radial fissure [4] may come into contact with the capillary network of the AF [48]. This would provide a direct route between blood circulation and NP tissue for circulating leukocyte entry into the intact IVD. Resolution without medical intervention has been described previously for protrusion-type IVD prolapse, and retraction of the AF is postulated to mediate this process [24,49]. Based on our observations, leukocyte-mediated breakdown of displaced NP tissue within the intact IVD could occur and may therefore represent a further mechanism in the process of protrusion resolution.

The context in which we sought to identify the cytokine and chemokine expression profile of the IVD was in so far as it related to the progression of IVD pathology, and to the effects that it may elicit on attempted biological therapies targeted at impeding progression of, or even reversing this pathology. In respect of IVD degeneration, pathological changes associated with altered cell behaviour are related to proliferation [5], differentiation [6-8], dysregulated metabolism [3,9] and cell death [10,11]. It seems reasonable to assume that if the cytokines and chemokines identified here do form part of the pathological mechanism of IVD degeneration and prolapse, their effects would be related to the adverse alterations in cell behaviour observed in IVD pathology.

Here, we attempted to systematically profile cytokine and chemokine expression in NP tissue. Initial screening of cytokine and chemokine gene expression in cells isolated from NP tissue specimens was performed by high sensitivity real-time q-PCR cDNA LDA. This method provides advantage over standard PCR detection because data collection within the exponential phase of amplification (prior to reaction saturation) makes this method fully quantifiable. Data generated therefore provide indication not just as to the levels of detection frequency in a study group, but also as to the relative expression levels within each study group. A limiting factor to this analysis, however, was the small number of samples subjected to LDA analysis. In light of the small study groups used, differential expression patterns observed between non-degenerate and degenerate study groups may not be representative of those in the wider population. Indeed, further real-time q-PCR analysis on larger study groups did not confirm all of the earlier LDA-indicated differential expression patterns.

Aside of the investigations described previously, the effects of cytokines and chemokines on NP cell behaviour have not been comprehensively studied, although their effects in other cell types have been described. Chondrocytes exhibit morphological and biochemical similarity to NP cells and so the effects of cytokines and chemokines on chondrocyte behaviour may be relevant to that of NP cells. In chondrocytes, effects of cytokine and chemokine stimulation have been described in relation to all of the common features of altered NP cell behaviour as seen in IVD degeneration. These include increased rates of proliferation [50], hypertrophic differentiation [51,52], increased catabolic metabolism [50,53,54], decreased anabolic metabolism [53,54], and induced cell death [55,56]. The precedents set in these investigations indicate that further investigative work into the role of cytokines and chemokines within the IVD is warranted.

Currently, several research initiatives are investigating the regenerative potential of cell therapy within the IVD [30-34]. Application of the soluble factor, transforming growth factor-β, elicits well-described effects on mesenchymal and adipose-derived stem cell differentiation; indeed, its application *in vitro* is required to stimulate differentiation towards an NP phenotype [31,35]. The survival and differentiation characteristics of stem cells injected into NP explant cultures, under conditions modelling the environment of the normal IVD have been confirmed [32]. However, the findings reported here suggest that cells implanted into the degenerate IVD would encounter a diverse and altered range of soluble factors, including cytokine and chemokine signalling molecules. The effects of these on stem cell behaviour have not been investigated and clearly the response of implanted cells to their surrounding environment will have considerable influence over the success of cell therapy to the IVD. To this end, these investigations provide insight into the conditions of the degenerate niche and indicate that further investigations to determine the effects this environment may elicit on cell behaviour are required.

## Conclusions

Our data indicate that NP cells are a source of cytokines and chemokines within the IVD and that these expression patterns are altered in IVD pathology. These findings may be important for the correct assessment of the degenerate niche prior to autologous or allogeneic cell transplantation for biological therapy of the degenerate IVD.

## Abbreviations

AF: Annulus fibrosus; CEP: Cartilaginous end plate; DMEM: Dulbecco’s modified Eagle's medium; ECM: Extracellular matrix; IHC: Immunohistochemistry; IL: Interleukin; IVD: intervertebral disc; LDA: low density array; LIF: leukaemia inhibitory factor; LIFR: Leukaemia inhibitory factor receptor; NP: Nucleus pulposus; PM: Post mortem; q-PCR: Quantitative polymerase chain reaction; TBS: Tris-buffered saline; TNF: Tumour necrosis factor.

## Competing interests

The authors declare that they have no competing interests.

## Authors’ contributions

KLEP performed all laboratory work, data analysis and statistical analysis, contributed to study design and drafted the manuscript. NC contributed to study design and sample collection, helped to secure funding and critically revised the manuscript. ALRM, AAC and LMB contributed to study design, sample collection and critically revised the manuscript. GH, RADB and AKC contributed to study design, helped to secure funding and critically revised the manuscript. CLLM conceived the study, participated in its design and coordination, secured funding and critically revised the manuscript for important intellectual content. All authors read and approved the final manuscript.

## Supplementary Material

Additional file 1 **Low density array (LDA) design.** (**I**) Internal reference and target genes investigated by cDNA LDA; green indicates internal reference genes. Highlighted genes are those selected for inclusion in additional real-time q-PCR analysis. (**II**) Catalogue numbers for pre-designed primer-probe sequences used in LDA analysis. Highlighted catalogue numbers are those for assays used in additional real-time q-PCR analysis. LDAs and primer-probe assays were supplied by Applied Biosystems (UK). Click here for file
