# Supplementary material for: The cytokine and chemokine expression profile of nucleus pulposus cells: implications for degeneration and regeneration of the intervertebral disc
Source: Arthritis Res Ther. 2013 Dec 11;15(6):R213. doi: 10.1186/ar4408 (PMC3979161; doi:10.1186/ar4408)
Supplement: Additional file 1 — Low density array (LDA) design. (I) Internal reference and target genes investigated by cDNA LDA; green indicates internal reference genes. Highlighted genes are those selected for inclusion in additional real-time q-PCR analysis. (II) Catalogue numbers for pre-designed primer-probe sequences used in LDA analysis. Highlighted catalogue numbers are those for assays used in additional real-time q-PCR analysis. LDAs and primer-probe assays were supplied by Applied Biosystems (UK). [file ar4408-S1.pdf]

(I)	1	2	3	4	5	6	7	8	9	10	11	12
A	**18S**	**GAPDH**	HPRT1	ACTB	PGK	IL-17RA	IL-22RαI	IFN-γRα	**gp130**	CXCL10	CCL19	CCR3
B	IL-1α	IL-15	IL-17F	CSF3	**IL-6R**	IL-17RB	IL-23R	IFN-γRβ	**CXCL1**	**CCL2**	CCL20	CCR5
C	**IL-1β**	**IL-16**	**IL-18**	IFN-γ	IL-7R	IL-17RC	IL-12Rβ1	OSMR	**CXCL2**	**CCL3**	**CX3CL1**	CCR6
D	IL-4	IL-17A	**IL-20**	**OSM**	IL-10Rα	IL-17RD	**TNF-R55**	**LIFRα**	**CXCL3**	**CCL4**	**CXCR1**	CCR7
E	**IL-6**	IL-17B	IL-23	**LIF**	IL-10Rβ	IL-18Rα	**TNF-R75**	IL-1Ra	CXCL5	**CCL5**	**CXCR2**	CCR8
F	IL-7	IL-17C	**TNF-α**	IL-1RI	IL-11Rα	IL-18Rβ	GM-CSFRα	IL-1RAcP	CXCL6	**CCL7**	CXCR3	**CX3CR1**
G	IL-10	**IL-17D**	TNF-β	IL-1RII	IL-15Rα	IL-20RI	GM-CSFRβ	ICE	**CXCL8**	CCL8	**CCR1**	Duffy
H	IL-11	IL-17E	CSF2	IL-4R	**CD4**	IL-20RII	G-CSFR	TACE	CXCL9	CCL13	**CCR2**	D6

(II)	1	2	3	4	5	6	7	8	9	10	11	12
A	**Hs99999901s1**	**Hs99999905m1**	Hs99999909m1	Hs99999903m1	Hs99999906m1	Hs01064648m1	Hs00222035m1	Hs00166223m1	**Hs01006727m1**	Hs00171042m1	Hs00171149m1	Hs00266213s1
B	Hs00174092m1	Hs00174106m1	Hs00369400m1	Hs99999083m1	**Hs01075667m1**	Hs00914532m1	Hs00332759m1	Hs00194264m1	**Hs00236937m1**	**Hs00234140m1**	Hs00171125m1	Hs00152917m1
C	**Hs00174097m1**	**Hs00189606m1**	**Hs00155517m1**	Hs00174143m1	Hs00902334m1	Hs00262062m1	Hs00538167m1	Hs00384278m1	**Hs00236966m1**	**Hs00234142m1**	**Hs00171086m1**	Hs00171121m1
D	Hs00174122m1	Hs00174383m1	**Hs00218888m1**	**Hs00171165m1**	Hs00387004m1	Hs00296982m1	**Hs01042313m1**	**Hs00158730m1**	**Hs00171061m1**	**Hs99999148m1**	**Hs00174146m1**	Hs01013469m1
E	**Hs00174131m1**	Hs00975262m1	Hs00372324m1	**Hs00171455m1**	Hs00175123m1	Hs00977691m1	**Hs00961755m1**	Hs00174099m1	Hs00171085m1	**Hs00174575m1**	**Hs00174304m1**	Hs00174764m1
F	Hs00174202m1	Hs00171163m1	**Hs00174128m1**	Hs00991002m1	Hs00234415m1	Hs00977695m1	Hs00538896m1	Hs00370506m1	Hs00237017m1	**Hs00171147m1**	Hs00171041m1	**Hs00365842m1**
G	Hs00174086m1	**Hs00370528m1**	Hs00236874m1	Hs00174759m1	Hs00542604m1	Hs00205346m1	Hs00166144m1	Hs00169146m1	**Hs00174103m1**	Hs00271615m1	**Hs00174298m1**	Hs01011079s1
H	Hs00174148m1	Hs00224471m1	Hs00171266m1	Hs00166237m1	**Hs00181217m1**	Hs00376373m1	Hs01114427m1	Hs00234224m1	Hs00171065m1	Hs00234646m1	**Hs00174150m1**	Hs00174299m1

Additional file 1. Low Density Array (LDA) Design

(I) Internal reference and target genes investigated by cDNA LDA, green indicates internal reference genes. Highlighted genes are those selected for inclusion in additional real-time q-PCR analysis.

(II) Catalogue numbers for pre-designed primer-probe sequences used in LDA analysis. Highlighted catalogue numbers are those for assays used in additional real-time q-PCR analysis. LDAs and primer-probe assays were supplied by Applied Biosystems (UK).
